# On the mechanism of performance improvement of electroactive polyvinyl chloride (PVC) gel actuators via conductive fillers

**DOI:** 10.1038/s41598-022-14188-9

**Published:** 2022-06-20

**Authors:** Zachary Frank, Kwang J. Kim

**Affiliations:** Active Materials and Smart Living Laboratory, Department of Mechanical Engineering, The University of Nevada, Las Vegas (UNLV), 4505 Maryland Parkway, Las Vegas, NV 89154-4027 USA

**Keywords:** Engineering, Materials science

## Abstract

The electromechanical actuation of transparent plasticized polyvinyl chloride (PVC) gels with conductive fillers were studied. The effects of functionalized carbon nanotubes (CNTs) and 1-butyl-3-methylimidazolium tetrafluoroborate ([Bmim]BF_4_) ionic liquid (IL) on both the electrical conduction and dielectric processes within PVC gels were investigated, and the differences between the two were clarified. Both CNTs and IL were shown to increase the conductivity of the gels and produce larger electromechanical transduction of a contraction actuator, but only CNTs were shown to increase the electrostatic adhesion force of the PVC gels. The addition of charge carriers to the gel via the inclusion of ILs was shown to significantly reduce the conductivity relaxation time, and the transient current upon voltage polarity reversal indicated multiple peaks corresponding to the introduction of carriers with different polarities and mobilities into the gel. This is believed to cause a screening effect, reducing the charge accumulation at the anode that is the foundational basis for PVC gels’ actuation mechanism. A recommendation for preferable conductive fillers for various applications is made.

## Introduction

Electroactive polymers (EAPs), polymer materials that convert electrical energy into mechanical work have been of interest to the research community for their possible use in a variety of applications in the field of soft robotics^1–4^. Dielectric elastomer actuators (DEAs) are one of the most common types of EAP within the field, but require multiple kilovolts for actuation^5–8^. PVC gels have been identified as a strong alternative for many applications, with PVC gels having much larger strains than DEAs at a given voltage and fast response rates, while still being more reliable and having faster response rates than other smart materials such as ionic polymer metal composites (IPMCs) and conductive polymers^9,10^. PVC gel actuators’ deformation is localized within a thin layer at the anode surface, allowing for unique actuation characteristics based upon the electrode configuration^11^. The actuation mechanism of PVC gels allows for a variety of actuator geometries including contraction, bending, and varifocal lenses among others^12–16^. While PVC gels offer great advantages over DEAs in terms of actuation voltage, their force production is quite low due to their inherently soft nature^17^. In addition, PVC gels still require higher voltages than what would be viable for many common applications (generally operating from 200 to 2 kV)^18^. While reduction of gel thickness can reduce this somewhat (as a function of increasing the electric field for a given applied voltage), additional advancements are still necessary. One area which might enable this advancement in terms of increased force production at lower voltages is through enhancement via fillers.

The addition of fillers to PVC gels has been shown to increase performance, with the most commonly studied additives being ionic liquids (ILs) and carbon-based fillers (CBFs) such as graphene oxide (GO) and carbon nanotubes (CNTs)^19–23^. These studies have been mostly focused on the characterization of actuator electromechanical properties of the materials, but little has been done to elucidate the differences in mechanism between ILs and solid CBFs, as well as whether one performs better than the other and under what circumstances. CBFs have been of particular interest given that multiple hypotheses for how they improve performance have been put forth. Either from interaction with the PVC polymer backbone leading to increased conductivity or through increased force output via electrophoretic effects^22,24^.

**Figure 1:**
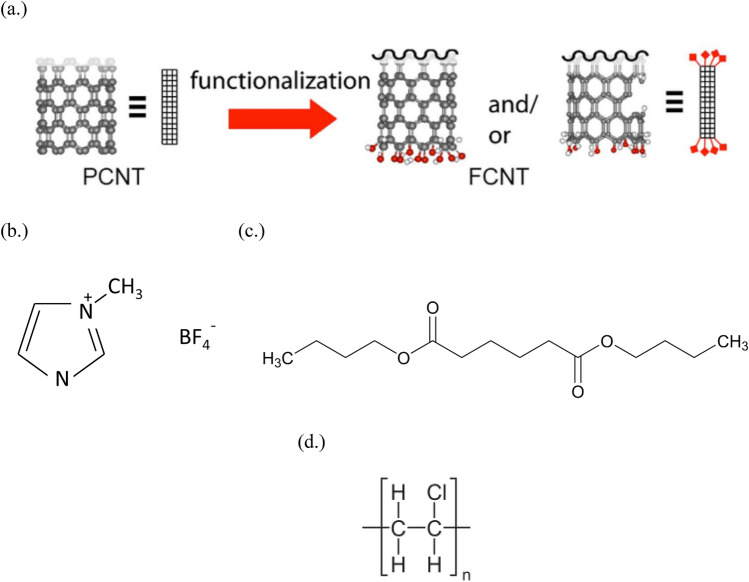
(**a**) Schematic illustration of functionalization process of carbon nanotubes; black, red, and white balls represent carbon, oxygen, and hydrogen, respectively. Image reused from^25^ with CC general permission. (**b**) Chemical structure of [Bmim]BF_4_. (**c**) Chemical structure of DBA. (**d**) Chemical structure of PVC.

Herein, we investigate PVC gels, plasticized with dibutyl adipate (DBA), and containing ILs (specifically, 1-butyl-3-methylimidazolium tetrafluoroborate ([Bmim]BF_4_), and CNTs. The functionalization process (addition of carboxyl and hydroxyl groups to pristine CNTs) for the CNTs and the chemical structure of the ILs, DBA plasticizer, and PVC used are shown in Fig. 1. Data for PVC gels with varying plasticizer content (1 part PVC to 1, 2, 4, 6, and 8 parts DBA, respectively) is also shown. We aim to provide a more detailed look at the charge transfer process that occurs with different conductive fillers through the analysis of their impedance, dielectric, and electric modulus spectra, as well as with current measurement under a reversal of applied voltage polarity. The electromechanical deformation and electrostatic adhesive force of PVC gels with ILs and CNTs are described. The differences in the mechanism between the two are clarified with a recommendation based upon application and desired characteristics. Table 1 summarizes the prepared PVC gel samples for the study.

**Table 1:** Summary of prepared PVC gels.

Label	PVC:DBA wt-% Ratio	Additive	Additive content wt-%
P4	1:4	N/A	N/A
P4-IL1	1:4	[Bmim]BF_4_	0.01%
P4-IL5	1:4	[Bmim]BF_4_	0.05%
P4-CNT2	1:4	Functionalized carbon nanotubes	0.02%
P1	1:1	N/A	N/A
P2	1:2	N/A	N/A
P6	1:6	N/A	N/A
P8	1:8	N/A	N/A

## Results and discussion

### Impedance spectra

**Figure 2:**
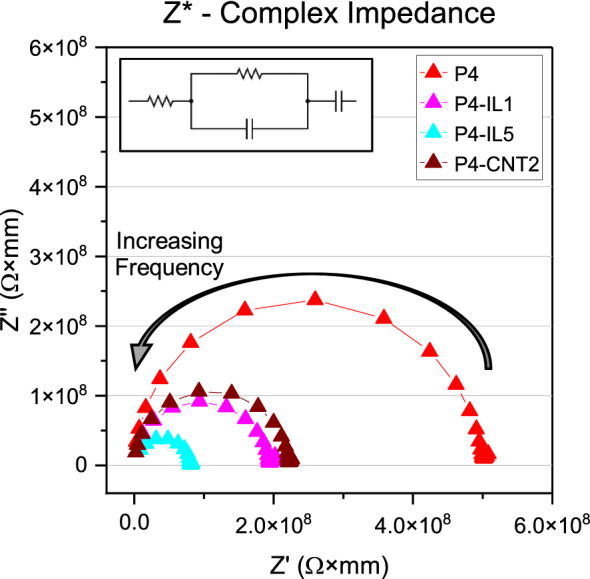
Argand plot of complex impedance of PVC gels with fillers.

The AC complex impedance (${Z}^{*}$) can be analyzed to show the conductivity and dielectric behavior of PVC gels with IL and carbon-based fillers. The Argand plot for PVC gels with varying additive content can be seen in Fig. 2. The ${Z}^{*}$ Argand plot consists of an inclined line at lower frequencies and a semicircle that intercepts the real axis at low and high frequencies. This characteristic of PVC gels can be represented by an equivalent circuit (Fig. 2 inset). The inclined lines in the ${Z}^{*}$ spectra correspond to an electrical double-layer capacitance at the interface of the gel and the electrodes and is represented as a capacitor in the equivalent circuit. The parallel capacitance and resistance in the equivalent circuit can be associated with the charge transport from the alternating electric field. A series resistance which represents the electrode resistance at very high frequencies is also shown and is the offset of the real impedance at high frequencies from ${Z}^{*}$ = 0; this element is low in comparison to the total impedance of the gels. The DC conductivity (*σ*_DC_) appears in the impedance spectra as the diameter of the semicircle formed between ${Z}^{*}$ and the low and high frequency intercept with the real axis in Fig. 2 and is listed along with other measured properties of PVC gels in Table 2. The conductivity can be seen to increase substantially with the addition of conductive filler, with ILs providing a greater decrease in impedance for a given filler quantity.

**Table 2:** A summary of electric properties of PVC gel samples.

Gel	$\upsigma _{{{\text{DC}}}}$(S/cm)	$\omega_{max}^{{M^{{\prime \prime }} }}$(rad/s)	$\omega_{max}^{\tan \delta }$(rad/s)	$\tan \left( \delta \right)_{max}$	$\tau_{EP}$(s)	$\varepsilon_{EP}$
P4	2.01** × **10^–8^	3.61** × **10^4^	220	50.7	0.66	1.43** × **10^5^
P4-IL1	8.22** × **10^–8^	8.30** × **10^4^	573	45.1	0.24	1.59** × **10^5^
P4-IL5	1.24** × **10^–7^	1.70** × **10^5^	734	79.6	0.32	4.80** × **10^5^
P4-CNT2	4.53** × **10^–8^	4.51** × **10^4^	306	49.0	0.44	2.28** × **10^5^
P2	5.25** × **10^–9^	2.09** × **10^3^	116	16.9	0.32	2.21** × **10^4^
P6	2.85** × **10^–8^	4.94** × **10^4^	331	54.0	0.48	1.68** × **10^5^
P8	3.84** × **10^–8^	5.80** × **10^4^	409	52.1	0.42	2.03** × **10^5^

### Permittivity and electric modulus spectra

**Figure 3:**
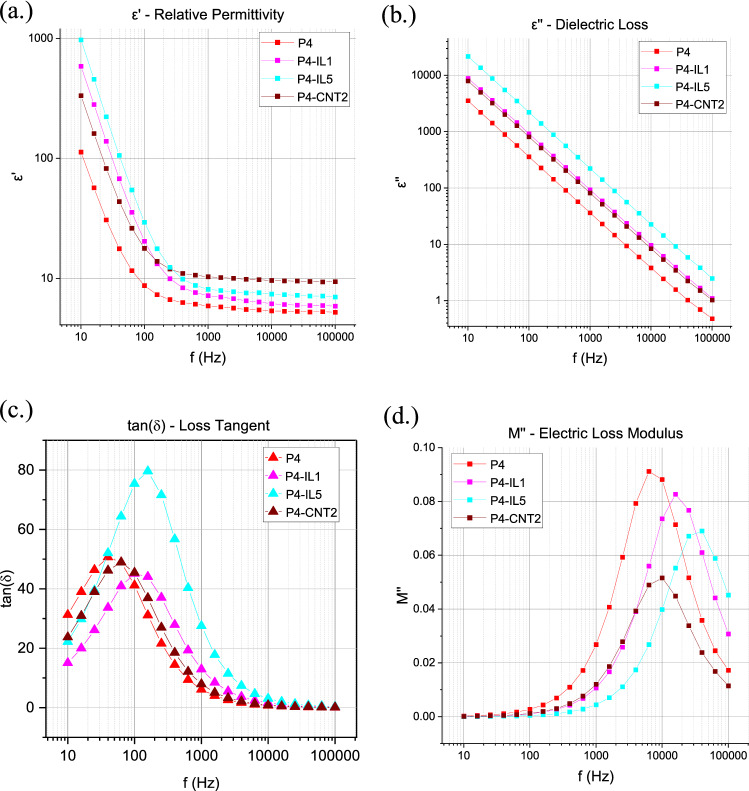
(**a**) Relative permittivity, (**b**) dielectric loss, (**c**) loss tangent comparison, (**d**) electric loss modulus M″ versus frequency comparison for different conductive fillers.

The complex permittivity ($\varepsilon^{*}$) of PVC gels consists of a real portion (relative permittivity, $\varepsilon ^{\prime}$) and an imaginary portion (dielectric loss, $\varepsilon ^{\prime\prime}$). $\varepsilon^{\prime}$ is the amount of charge stored via dipole interactions with the electric field, while $\varepsilon ^{\prime\prime}$ is the energy lost due to the resistance of dipoles to rotation in the alternating electric field and the conduction of charge as a result of the applied field. Both ${{\upvarepsilon^{\prime}}}$ and ${{\upvarepsilon^{\prime\prime}}}$ can be found from $Z^{*}$ through the relationships in Eq. (1):

1 $$ \varepsilon^{*} = \varepsilon^{\prime} - j\varepsilon^{\prime\prime} = \left( {\frac{{Z^{\prime\prime}}}{{\omega C_{0} \left| Z \right|^{2} }}} \right) - j\left( {\frac{{Z^{\prime}}}{{\omega C_{0} \left| Z \right|^{2} }}} \right) $$

where $\omega$ is the angular frequency ($\omega = 2\pi f$), and $C_{0}$ is the capacitance of free space ($C_{0} = \frac{{{\upvarepsilon }_{0} A}}{d}$; $A$ and $d$ are the electrode contact area and gel thickness, respectively.). The comparison of relative permittivity and dielectric loss for PVC gels with different additive content can be seen in Fig. 3a,b, respectively, along with the loss tangent ($\mathrm{tan}\left(\delta \right)={\varepsilon }^{{^{\prime}}{^{\prime}}}/{\varepsilon }^{^{\prime}}$) which is shown in Fig. 3c.

The C–Cl bonds in the PVC are dipolar, as are the Carboalkoxy (RCOOR′) functional group in the adipate plasticizer. The increase in the free volume of the gel allows for greater polarizability of the C–Cl bonds, and thus PVC gels have a higher relative permittivity than unplasticized PVC at all frequencies^26^. The permittivity of the PVC gels dielectric spectra shows low-frequency dispersion, where the relative permittivity can be seen to increase at low frequencies caused by electrode polarization (EP)^27^. The EP is a result of charge motion through the gel. This behavior is specifically tied to the effects of the added liquid phase plasticizer to the PVC matrix, as it is not seen in PVC or DBA alone^28^.

The inclusion of conductive fillers, both CNTs and [Bmim]BF_4_ ionic liquid increased the permittivity and dielectric loss within the PVC gels. The dielectric permittivity at high frequencies (which is indicative of the “true” relative permittivity, i.e., without the effect of EP) was largest for the PVC gel with CNTs, indicating that the overall polarizability of the gels is higher with CNTs. By contrast, only a small increase in relative permittivity is seen in the case of PVC gels with ILs at high frequencies. At lower frequencies, the ILs have a much higher permittivity than the unmodified or CNT gels indicating that the addition of charge carriers lead to a reduced Debye length in the gels with ILs. While CNTs have additional charge carriers (such as holes and electrons), the gels are primarily ionic conductors. The solvating ester groups of the plasticizer induce motion of the gel with the space charge formed from impurity ions within the pure PVC-plasticizer material as well as with the additional ions from the ILs.

The complex-valued electric modulus is:

2 $$ M^{*} = \frac{1}{{\varepsilon^{*} }} = \frac{{\varepsilon^{\prime}}}{{(\varepsilon^{{\prime}{2}} + \varepsilon^{{\prime\prime}{2}} )}} + \frac{{j\varepsilon^{\prime\prime}}}{{(\varepsilon^{{\prime}{2}} + \varepsilon^{{\prime\prime}{2}} )}} = M^{\prime} + jM^{\prime\prime} $$

with real ($M^{\prime}$) and imaginary ($M^{\prime\prime}$, or electric loss modulus) components. The conductivity relaxation frequency ($\omega_{m}$) at which the peak of $M^{\prime\prime}$ occurs, gives the most probable conductivity relaxation time ($\tau_{M}$)^29^. When $f > f_{max}^{{Z^{\prime\prime}}}$ ,the charge transport occurs only via local short-range motion, as the charges move via trap-controlled hopping between localized potential wells, and the dipoles in the gel are unable to reorient themselves before the polarity of field reverses^30^. When the frequency drops below $f_{max}^{{M^{\prime\prime}}}$ the charges have time to move between the electrodes (conductivity relaxation). $M^{\prime\prime}$ can be related to the long-range electric relaxation of the material, while $Z^{\prime\prime}$ is related to the localized relaxation of dipoles.

3 $$ M^{\prime\prime} = Z^{\prime}\left( {\frac{{\omega \varepsilon_{0} A}}{d}} \right) $$

4 $$ Z^{\prime\prime} = \varepsilon^{\prime}\left| Z \right|^{2} \left( {\frac{{\omega \varepsilon_{0} A}}{d}} \right) $$

With the results presented in Fig. 3d we can say more about the differences between these two types of fillers (CBFs and ILs), and how they contribute to the electromechanical transduction in PVC gels. In the case of CNTs, the peak of $M^{\prime\prime}$ can be seen to decrease significantly, indicating a lower real impedance value with increasing filler content; however, only a small shift in the peak frequency is seen. This suggests that the relaxation of mobile charges within the CNT gels occurs over a similar timeframe, but the real impedance of the gels is significantly reduced. This can be explained by the fact that the resistivity of the gel is lowered with filler content as ionic conductivity is enhanced via increased ionic mobility for the same applied voltage^23,24^. By contrast, ILs both decreased the ${M}^{{^{\prime}}{^{\prime}}}$ peak and shifted the spectra towards higher frequencies. This indicates that the ILs increase the conductivity of the gels, and the long-ranging electric charges have a faster relaxation than the charges present in unmodified gels alone. The ions are able to dissociate and relax at higher frequencies than the ions and injected charges present in the unmodified gels alone. This leads to ILs having slightly larger deformation, but much faster actuation at lower voltages as reported upon previously by multiple other groups^19–21^. The electrode polarization time and maximum apparent permittivity are found through the fitting of a Cole–Cole equation to the tan(*δ*) spectra of the gels and can be seen in Table 2.

### Polarity reversal response and mobility measurement

**Figure 4:**
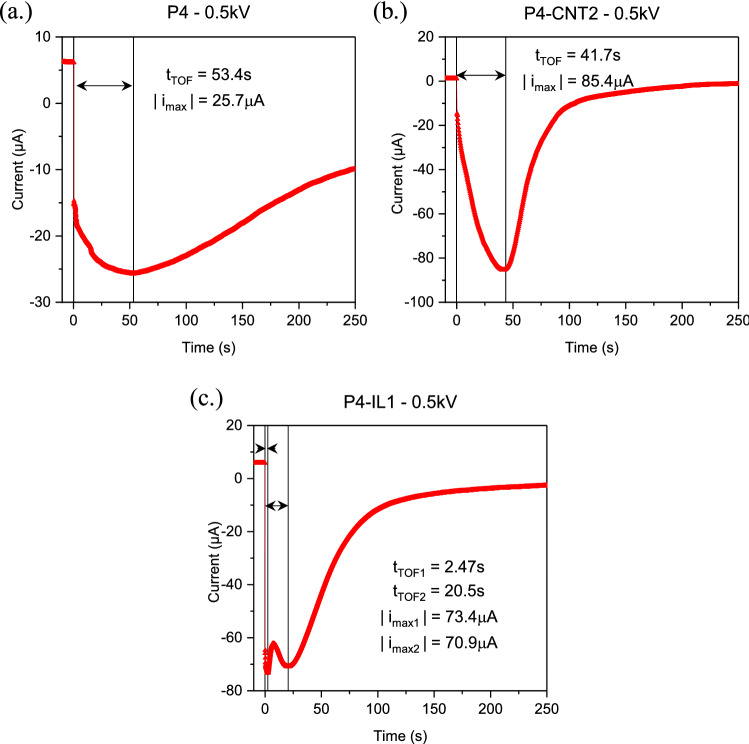
(**a**) i-t plot for a 0.5 kV voltage polarity reversal in P4, (**b**) P4-CNT2, and (**c**) P4-IL1.

A DC electric field was applied to the PVC gel and polarization was allowed to saturate (t > 15 min); the electric current decreased to a low steady-state value (leakage current). Upon reversal of the polarity of the field, the current in the PVC gels can be seen to increase as the charges are moved away from the electrode and towards the new anode. The time for the peak current in the gel after polarity reversal gives a time-of-flight (TOF) for the charge carriers to move across the gel. This TOF measurement ($t_{TOF}$) has previously been used to calculate the mobility of charge carriers within a material. However, many different models have been created based upon the type of charge carrier, thickness of the polymer, polymer material, the electric field magnitude, and other factors^31–33^. Given the disagreement in the field on whether polarity reversal can be used to determine the true mobility of charge carriers, for the purpose of this study the determination of the exact carrier mobility is not concluded, and instead we will refer to the calculated value as the “apparent mobility” $\left( {\mu_{ap.} } \right)$. The model developed by Many and Rakavy, which includes cathode electron injection space charge effects (which has previously been used to describe the transient current peak within plasticized PVC containing absorbed water) will be used to inform our interpretation $\left( {\mu_{ap.} = 0.786\frac{{d^{2} }}{{t_{TOF} V}}} \right)$^33,34^. There are two primary takeaways from Fig. 4a–c. The first is that a double peak can be seen in P4-IL1 gels, suggesting that multiple charge carriers are present^31,35^. The apparent mobility of the first peak is nearly an order of magnitude larger than the second ($\mu_{ap.} = 7.7 \times 10^{ - 6}$ cm^2^/V s, versus $9.3 \times 10^{ - 7}$ cm^2^/V s). The presence of multiple charge carriers in this measurement is not unexpected, as clearly ILs consist of both an anionic ([BF_4_] in our case) and cationic ([Bmim]) component. The presence of a second charge carrier though means that some of the electric field drop which occurs in the space charge region will be screened by the double layer at the cathode interface leading to a lower overall space charge density. In fact, this has been previously reported on through space charge density measurements, and also is likely the cause of the lower electrostatic adhesive force for P4-IL1 gels seen in the next section^20^. The apparent mobilities of carriers in P4-IL1 is much higher than that of either P4 (1.24** × **10^–7^ cm^2^/V s) or P4-CNT2 (2.41** × **10^–7^ cm^2^/V s). Additionally, we can use the apparent mobility obtained here to compare the implied change in conductivity and the DC conductivity as measured from impedance spectroscopy. Conductivity is proportional to both the number of charge carriers and the mobility of those charge carriers ($\sigma = nq\mu$). Comparing the increase in conductivity between P4 and P4-CNT2, the value from the high voltage polarity reversal is only 13% lower than that from the low voltage impedance measurement. This implies that the change in conductivity is most likely due to the increased ionic mobility of the material with added CNTs. In the case of P4-IL1 however, we can see that the mobility associated with both charge carriers increases substantially more than the conductivity. In addition, the number of charge carriers should also have increased, as both positive and negative carriers were added in the form of IL. Given this, the conclusion can be made that the mobility of the charges inherent to unmodified PVC gels was significantly reduced, or complex interactions between the bulk gel and ionic dissociation resulted in a reduced number of total carriers in the gel.

### Actuation and electrostatic adhesion force

**Figure 5:**
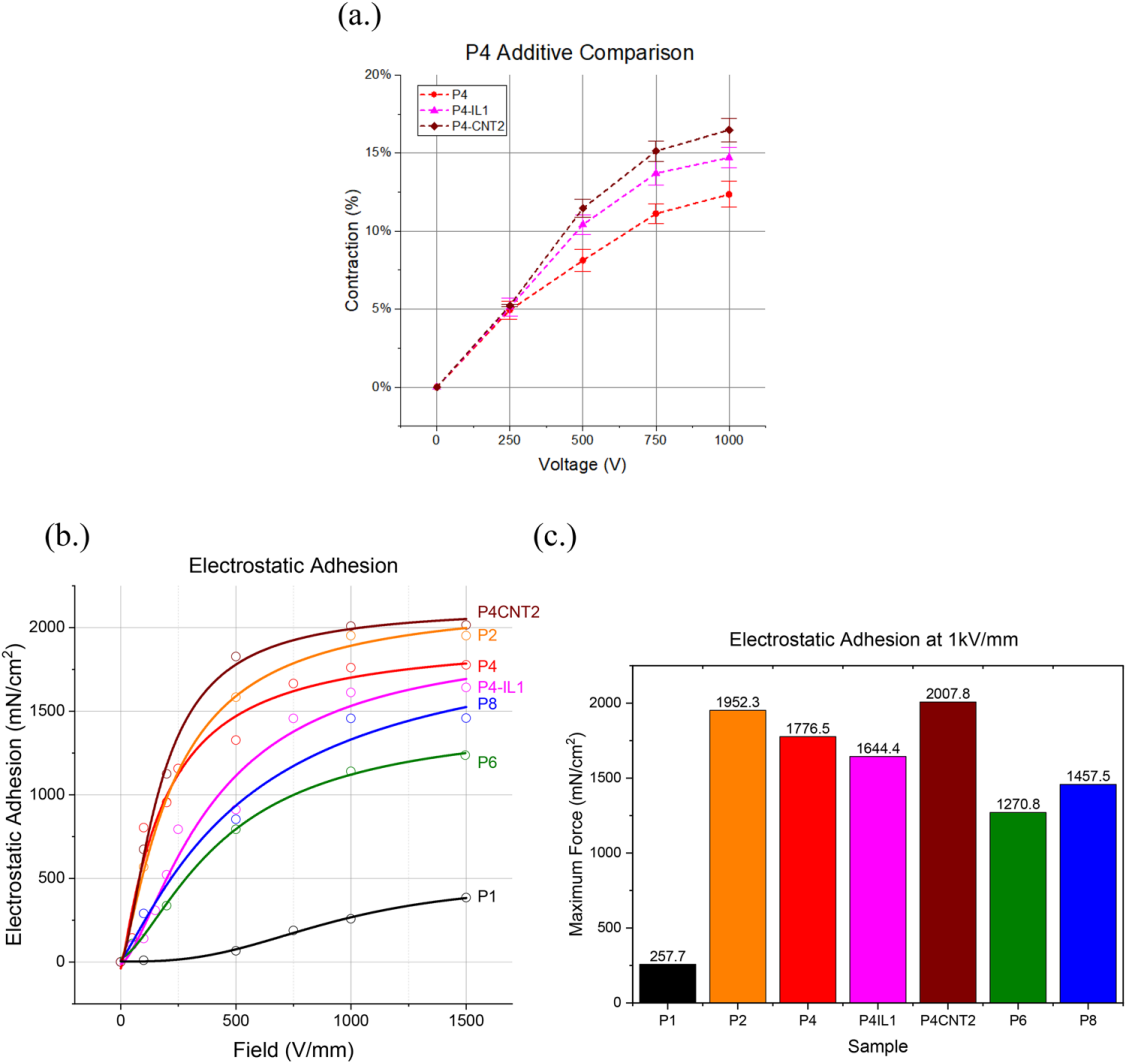
(**a**) Contraction of a mesh actuator for gels with different additive content. (**b**) Electrostatic adhesion force at 1 kV/mm for PVC gels with different filler and plasticizer content. (**c**) Electrostatic adhesion force for varying electric field intensity.

The deformation of P4, P4-IL1, and P4-CNT2 was measured with increasing voltage P4-CNT2 can be seen from Fig. 5a to have the largest displacement of the three with substantial increased contraction over P4 with the same electrode configuration. P4-IL1 showed improvement over the unmodified gel as well, both results are consistent with previous studies showing better actuation performance for gels with conductive fillers^19,21,23^.

The accumulation of negative charges within the PVC gels near the anode creates an attractive force to the opposing positive charges on the conductive electrode surface. The electrostatic adhesive force only occurs on the anode due to the asymmetric formation of space charge near it when compared to the cathode (the measured electrostatic force on the cathode was zero for all samples). PVC gels have an initial tackiness that increases with plasticizer content and has been subtracted from the overall force measured in the electrostatic adhesion measurements shown in Fig. 5b,c. Due to the unique asymmetric space charge in PVC gels the electrostatic adhesive force must be measured differently than for common electrostatic adhesion actuators (where the electrostatic adhesion occurs at both the electrodes and at non-conductive contact surfaces). This was measured using a method similar to that used by Ali et al. in their paper on the measurement of electrostatic adhesion force of PVC gels with varying plasticizer content^28^. The gels were fixed to the cathode using a 3D printed gel holder (Figure S2), with a slot for anode attachment and a fixture to allow for the addition of mass to the anode. After a voltage was applied, the mass was then lifted at 1 mm/s, with repetition at higher masses until the electrostatic adhesion of the gel was no longer able to hold the mass. All gels tested showed an initial large increase in electrostatic adhesion followed by a plateau at higher voltages which is linked to the leakage current of the gels and is common among electrostatic adhesion actuators^36^. The IL-doped gels saw a net reduction of electrostatic force production, which corresponds to reports of lower overall space charge in IL gels^20^. CNTs by contrast, saw an improvement in the electrostatic adhesion over pure PVC gels. The CBFs appear to give larger displacement and faster response times, in addition to increased electrostatic force production and all at a higher material modulus indicates that they may be preferable over ionic liquids in most actuation applications^21–23^. One caveat to this takeaway is that ILs do increase the transparency of the PVC gel, while CBFs, by their nature, absorb visible light (Images of PVC gels and UV–Vis spectrum of P4 and P4-IL1 gels can be seen Supplementary Figures S3 and S4, respectively)^21^. This indicates that ILs may be uniquely suited as performance-enhancing additives for PVC gels in optical applications, which is a primary area of research in the field, and one of the most promising.

## Conclusion

The conductivity and dielectric characteristics of PVC gels with both IL and CNT fillers were studied, and the mechanisms of performance enhancement for each were clarified. CNTs improve the conductivity of the gels through the facilitation of charge transfer processes (increased mobility). This leads to faster polarization and increased actuator performance. ILs increased the number of carriers in the gel, through the introduction of anions and cations. While this increased the conductivity, a field screening effect was seen in the comparison of the implied conductivity from the apparent mobility (as determined by alternating polarity i-t measurements in “Polarity reversal response and mobility measurement” section) with the conductivity measured through impedance spectroscopy. This leads to a lower electric field in the space charge layer which is the primary mechanism of actuation in PVC gel. Due to this, ILs developed a lower electrostatic adhesion strength than the unmodified and CNT samples. While both CNTs and ILs mechanisms increase the displacement of the gels, CNTs do so while also improving the mechanical characteristics of the gels (increased modulus) and electrostatic force production. Additionally, the overall deformation was higher for CNTs than IL gels. This means that for many applications CBFs will be preferable over ILs, due their improved displacement and force production. In addition, ILs often have issues with leakage (migration of the ILs out of the host polymer), which could lead to degradation of performance over time or environmental contamination depending on the application. [Bmim]BF_4_, in particular has been shown to have issues with instability in the presence of water^37^. However, ILs still have specific areas where they excel, such as in optical applications which rely upon the transparency of PVC gels which is improved by ILs and is partially lost with the addition of CBFs.

## Methods

### Materials and preparation of PVC gels

PVC gels were made from polyvinyl chloride (M_w_ = 233,000 g mol^−1^, M_n_ = 99,000 g mol^−1^) in powder form, dibutyl adipate (DBA) (M_W_ = 258.38 g mol^−1^) plasticizer, and tetrahydrofuran (THF) solvent (20 ml solvent/gram of PVC), all of which were purchased from Sigma-Aldrich Co. The solution was then stirred for 24 h at 30 °C before being cast in a glass petri dish (10 cm diameter), where the THF was evaporated off over 4 days at room temperature. The gels were prepared by mixing 1-part PVC with X-parts DBA plasticizer by weight (gels are referred to as PX, with X indicating plasticizer content, e.g. P4, P6, P8, etc.). The gels produced ranged in thickness from 0.65 to 0.72 mm.

### Materials and preparation of PVC-IL gels

The IL used in the study was 1-Butyl-3-methylimidazolium tetrafluoroborate ([Bmim]BF_4_) and was also purchased from Sigma-Aldrich Co. Both additives (ILs and CNTs) were added in increments of 0.01%, and will be referred to by the PVC gel in which they are added followed by the amount of additive (e.g. P4 gel with 0.02% CNTs will be referred to as P4-CNT2). The ILs were mixed with the DBA and stirred for 30 min at room temperature before adding to the PVC and THF. The gels were then prepared in the same method as for unmodified PVC gels described above.

### Materials and preparation of functionalized CNTs and PVC-CNT gels

Pristine Carbon Nanotubes (unfunctionalized) were washed with HCl to remove impurities (2 g in 200 ml HCl). The mixture was stirred for 2 h, before being diluted with deionized (DI) water and dried in a convection oven for 24 h at 90 °C. For the functionalized CNTs, 0.5 g of purified CNTs were treated in an acid solution (60 ml H_2_SO_4_, 20 ml HNO_3_, and 1 g KMnO_4_) with a condenser where the liquid was refluxed for 8 h. The resulting liquid was diluted with DI water, filtered, and then washed to obtain a neutral pH before being dried again at 90 °C for 24 h. The CNTs were added to tetrahydrofuran (THF) (100 ppm by weight). The mixture was then dispersed via ultrasonication (VC-505, Sonics) for 3 h. After ultrasonication, the PVC and DBA were then immediately added to the CNT-THF mixture and prepared using the same method as the unmodified gels. A full list of gels used in this study with corresponding plasticizer and additive ratios is located in Table 1.

### Characterization

The conductivity and dielectric properties of the PVC gels were determined through the use of impedance spectroscopy. Measurements were taken with an LCR meter (ET4510, East Tester) with a 1 V amplitude alternating current (AC) signal, from 10 Hz to 100 kHz (5 points/decade). The samples (diameter of 30 mm) were placed between two 25.4 mm stainless steel parallel plate electrodes, and a load cell ensured consistent contact force across tests. The thickness of the gels shown were ~ 0.7 mm for the unmodified gels, 0.8 mm for the P4CNT2 and 1.1 mm for the IL gels. Multiple thicknesses were also tested with little variation in relative permittivity or dielectric loss between them.

For current and displacement tests, the voltage was produced with a signal generator (SDG-1025, Siglent Technologies), and a high voltage amplifier (609E-6, TRek). The response was analyzed with a data acquisition system (DAQ-6510, Keithley Instruments), and load cell (GSO-100, Transducer Techniques) which ensured consistent force across samples (Supplementary S2). A copper foil cathode and a stainless-steel (SS 304) mesh (size of 20 wires/inch) was used. The dimensions of the actuators used were 20 mm × 5 mm × 1.7 mm (length x width x thickness) for all gels. All measurements were taken at room temperature. A laser displacement sensor was used to measure the displacement of the actuators (optoNCDT-1401, Micro-Epsilon).

The electrostatic adhesive force is measured as the force of detachment between the PVC gel and the electrode. The cathode experiences nearly zero adhesion beyond the initial contact stickiness tackiness of the PVC gel (with zero applied voltage). As such, consistent contact with the cathode is necessary to maintain the adhesive force at the anode. A 3D printed mass holder was made which ensures consistent contact between the gel and the cathode, while also allowing for addition of increasing mass. The mass is lifted at 1 mm/s, and when the gel can no longer hold the mass (i.e. the mass is dropped), the peak force from the load sensor is recorded as the peak electrostatic force.

## Supplementary Information

Supplementary Figures.
