# Supplementary material for: On the mechanism of performance improvement of electroactive polyvinyl chloride (PVC) gel actuators via conductive fillers
Source: Sci Rep. 2022 Jun 20;12:10316. doi: 10.1038/s41598-022-14188-9 (PMC9209489; doi:10.1038/s41598-022-14188-9)
Supplement: Supplementary file 1 — Supplementary Figures. [file 41598_2022_14188_MOESM1_ESM.docx]

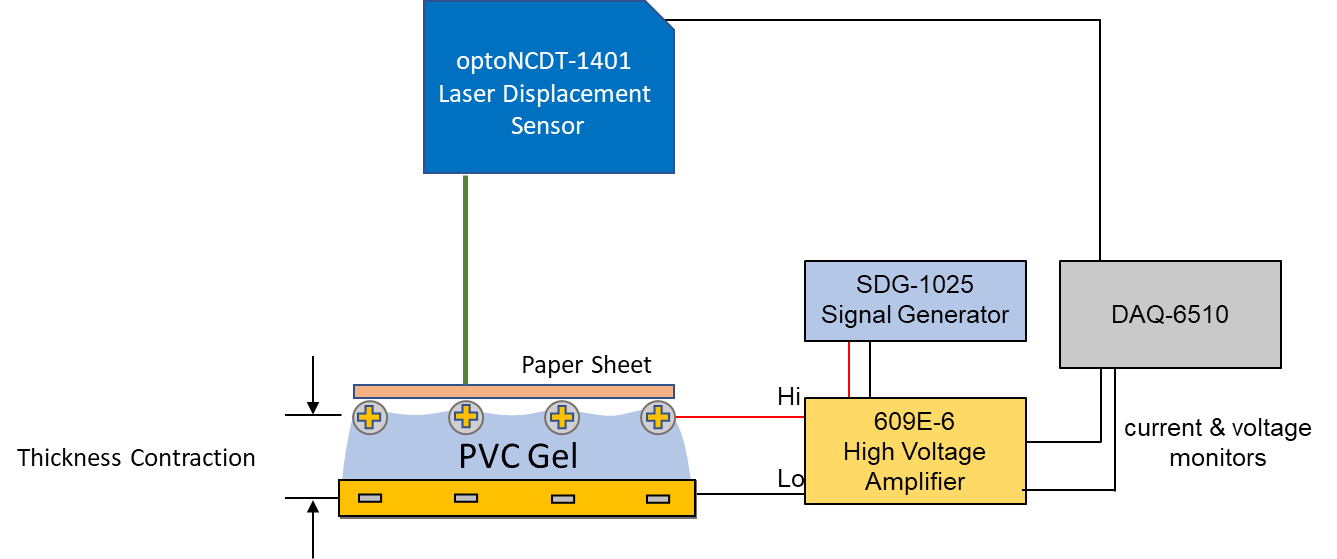


*Figure S1: Setup for PVC gel contraction actuator displacement test*


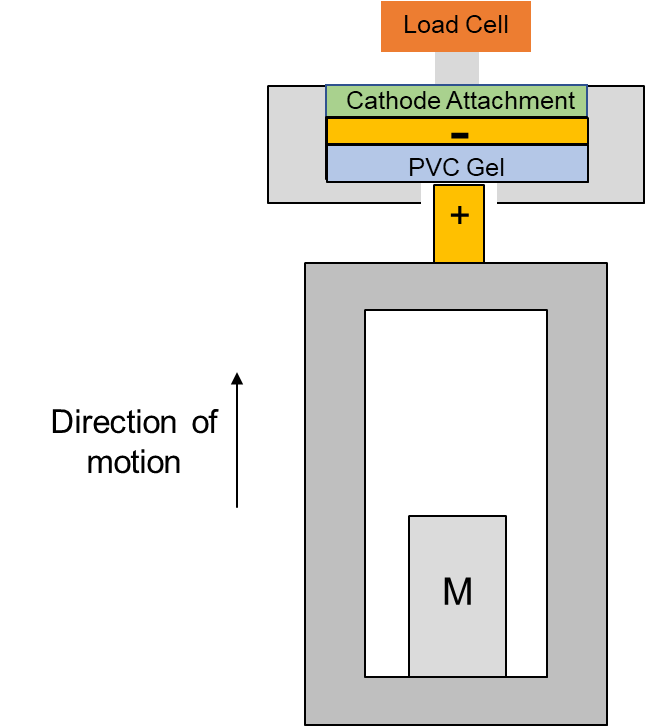

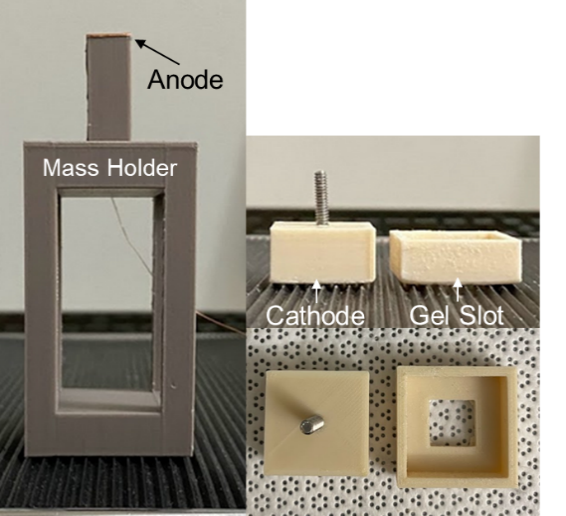


*Figure S2: PVC gel electrostatic adhesion test setup*


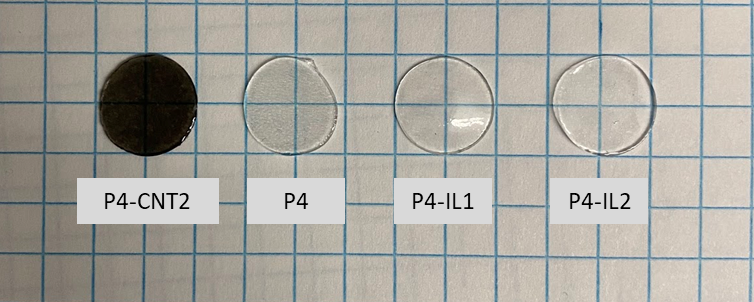


Figure S3: P4 gels with CNTs (left), unmodified (left-middle), 0.01% [Bmim]BF_4_ (right-middle) and 0.05% [Bmim]BF_4_ (right).

Figure S4: P4 and P4-IL1 optical transmission comparison (measured through UV-Vis spectroscopy).

(a.)

(b.) (c.)

Figure S5: Representative sample data of contraction of (a) P4, (b) P4IL1, and (c) P4CNT2 gel actuators in response to a 1kV electric field.

(a.)


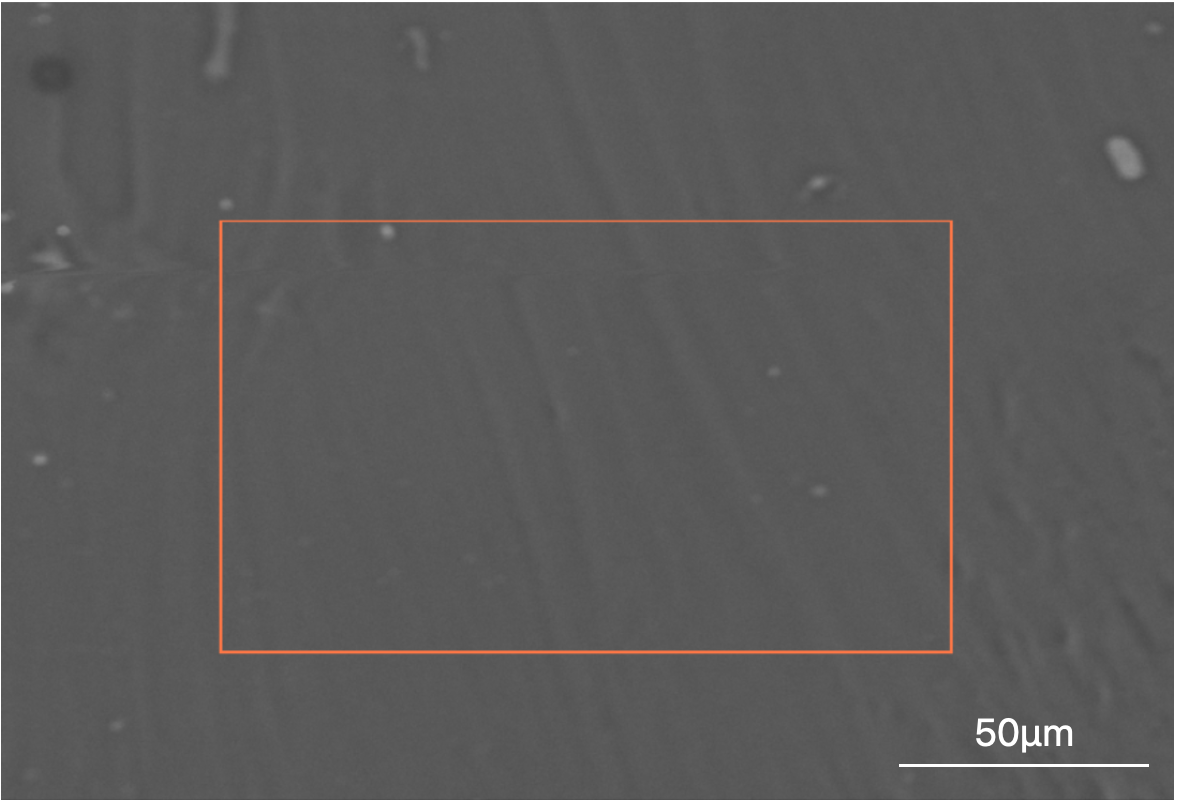

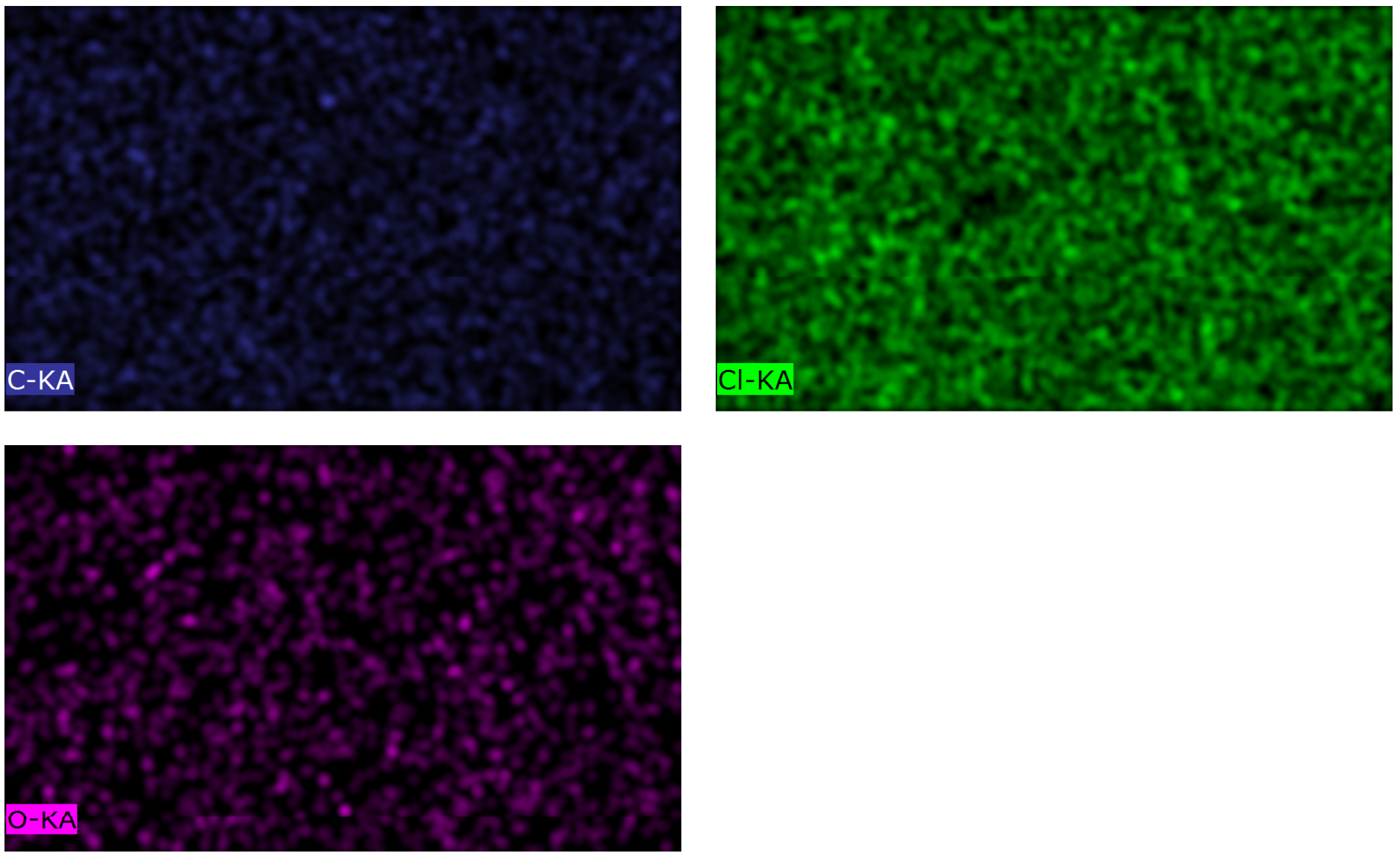


(b.)


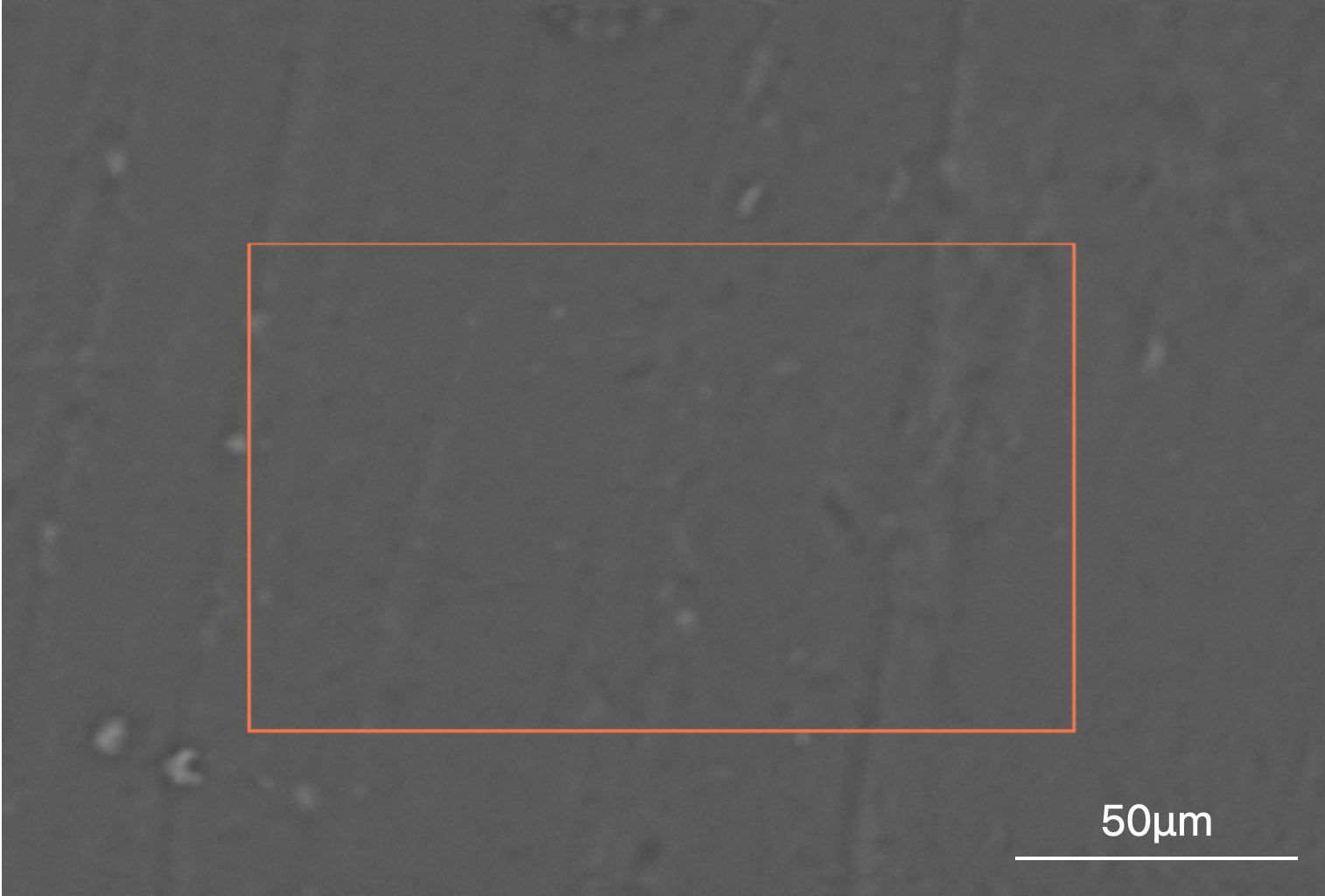

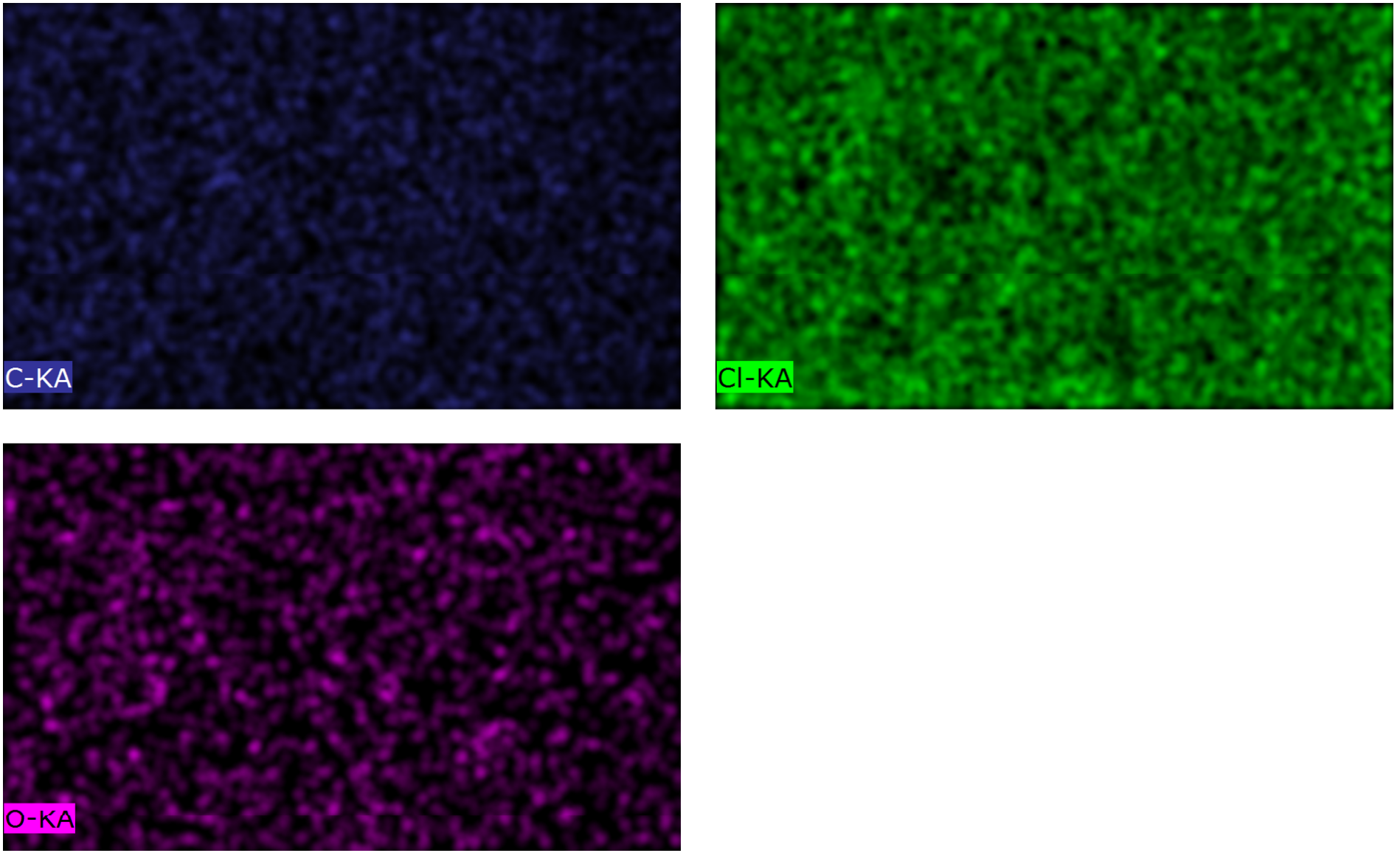


(c.)


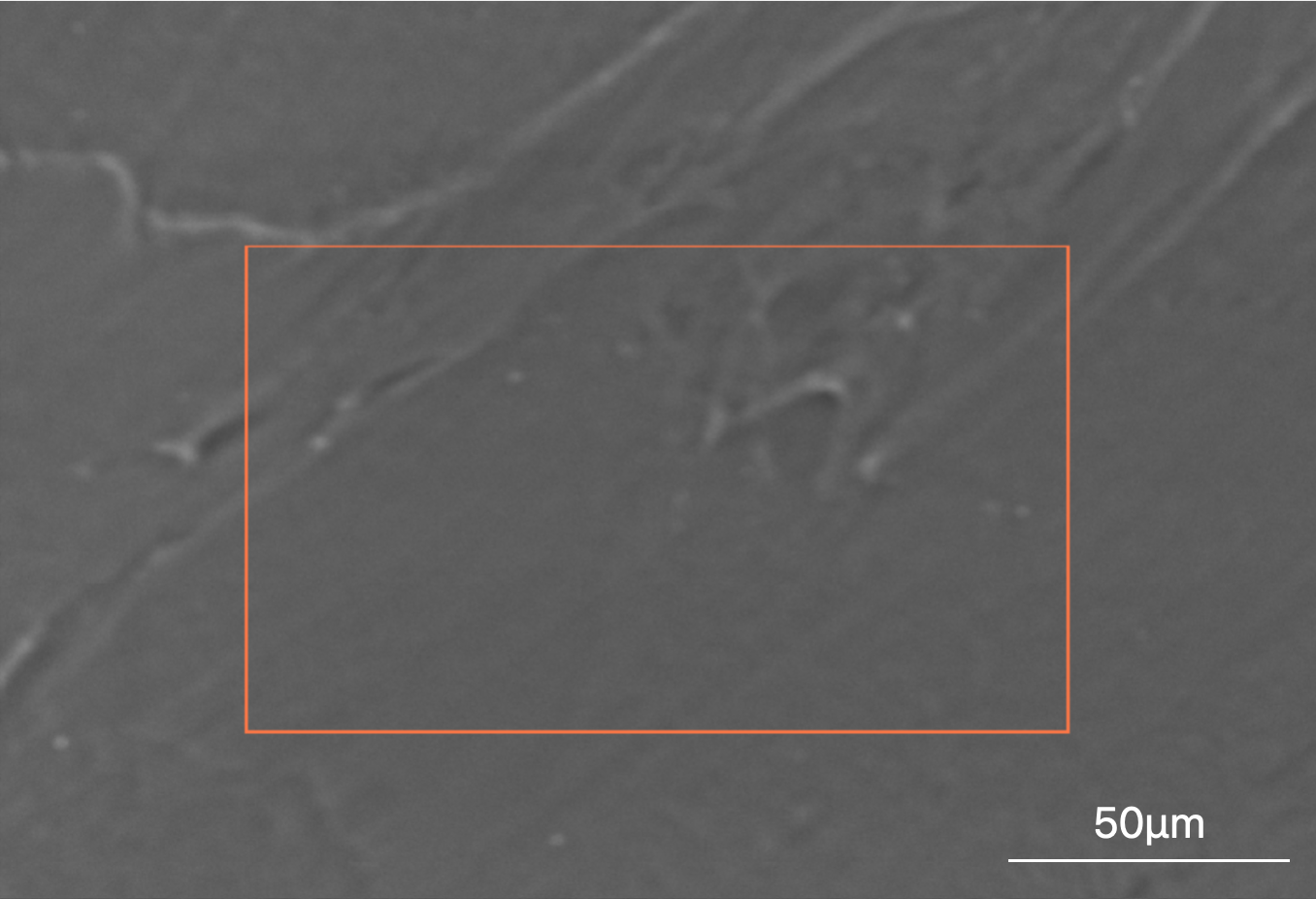

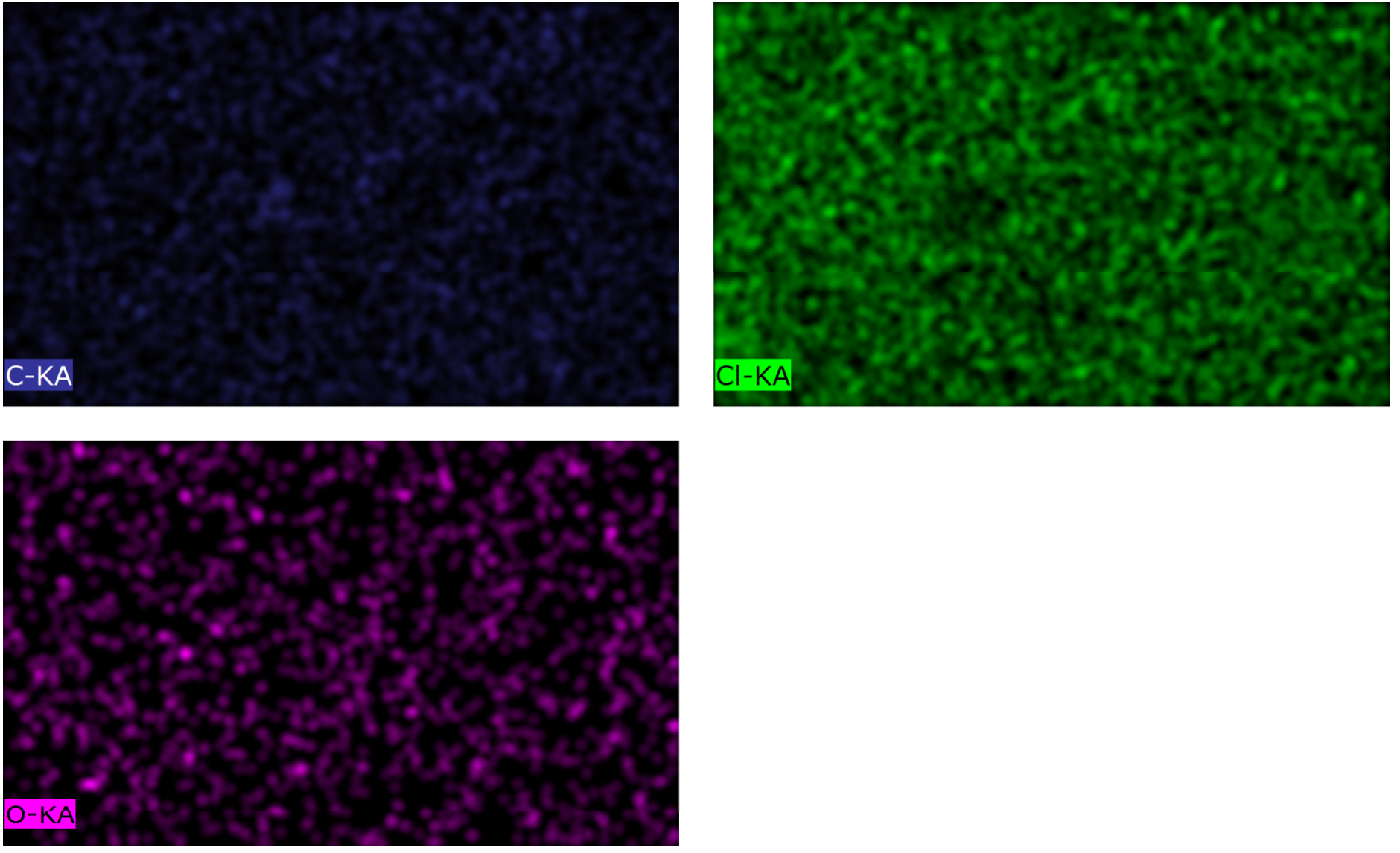


*Figure S5: SEM and EDS image of (a) P4, (b) P4IL1, and (c) P4CNT2 gels. No agglomeration was detected in the CNT containing gel.*
